# Suspected Germline TP53 Variants and Clonal Hematopoiesis of Indeterminate Potential: Lessons Learned From a Molecular Tumor Board

**DOI:** 10.1093/oncolo/oyad105

**Published:** 2023-05-09

**Authors:** Camila B Xavier, Rudinei Link, Leonília Abreu, Fabiana Bettoni, Fabiane Marson, Pedro A F Galante, Cibele Masotti, Mariane T Amano, Vinicius de Molla, Anamaria A Camargo, Paula F Asprino, Jorge Sabbaga

**Affiliations:** Oncology Center, Hospital Sírio-Libanês, São Paulo, SP, Brazil; Oncology Center, Hospital Sírio-Libanês, São Paulo, SP, Brazil; Molecular Oncology Center, Hospital Sírio-Libanês, São Paulo, SP, Brazil; Molecular Oncology Center, Hospital Sírio-Libanês, São Paulo, SP, Brazil; Oncology Center, Hospital Sírio-Libanês, São Paulo, SP, Brazil; Molecular Oncology Center, Hospital Sírio-Libanês, São Paulo, SP, Brazil; Molecular Oncology Center, Hospital Sírio-Libanês, São Paulo, SP, Brazil; Molecular Oncology Center, Hospital Sírio-Libanês, São Paulo, SP, Brazil; Hematology Center, Hospital Sírio-Libanês, São Paulo, SP, Brazil; Molecular Oncology Center, Hospital Sírio-Libanês, São Paulo, SP, Brazil; Molecular Oncology Center, Hospital Sírio-Libanês, São Paulo, SP, Brazil; Oncology Center, Hospital Sírio-Libanês, São Paulo, SP, Brazil; Instituto do Câncer do Estado de São Paulo, Universidade de São Paulo, São Paulo, SP, Brazil

## Abstract

**Objective:**

Li-Fraumeni syndrome (LFS) is a pan-cancer predisposition syndrome caused by germline pathogenic variants in the gene *TP53*. The interpretation of *TP53* variants in clinical scenarios outside the classic LFS criteria may be challenging. Here, we report a patient affected by 2 primary cancers at later ages, who harbored a likely pathogenic *TP53* at low allele frequency detected in a blood sample.

**Methods:**

The Molecular Tumor Board committee at our institution revisited the case of a patient who was enrolled in a research protocol for the investigation of genetic conditions associated with neuroendocrine tumors. Clinical, familial, and molecular data were reviewed. The patient received germline testing using a next generation sequencing multi-gene panel and was incidentally found to harbor a *TP53* likely pathogenic variant, with 22% of variant allele fraction. Additional samples, including a second blood sample, oral swab, and saliva, were collected for DNA analysis. A new *TP53* sequencing round was performed with the attempt to distinguish between a true constitutional germline variant and a somatically acquired variant due to aberrant clonal expansion of bone marrow precursors.

**Results:**

Patient’s personal and familial history of cancer did not meet classic nor Chompret LFS criteria. Environmental risk factors for cancer were identified, such as alcohol abuse and tobacco exposure. The TP53 variant initially found in the next-generation sequencing was confirmed by Sanger sequencing in the previous DNA sample extracted from blood for the first analysis and in a second blood sample collected 6 years later. The TP53 variant was not detected in the DNA extracted from the oral swab and saliva samples.

**Conclusion:**

Considering the low TP53 variant allele fraction in blood, absence of variant detection in oral swab and saliva samples, the lack of LFS clinical criteria, and history of exposure to environmental risk factors for cancer, the main hypothesis for this case was aberrant clonal expansion due to clonal hematopoiesis. Oncologists should interpret *TP53* findings during germline testing with caution.

## Teaser

This case report describes a patient with a history of 2 primary cancers who harbored a likely pathogenic TP53 variant with low variant allele frequency in a blood sample. Such a result could have been misinterpreted as a Li-Fraumeni case, but other tests were conducted to elucidate the case.

**Key Points**

- *TP53* pathogenic variants with low variant allele fraction identified during germline testing must be interpreted with caution.
- Complex multi-tissue analysis may be necessary to distinguish between constitutional and somatically acquired *TP53* pathogenic variants with low variant allele fraction. This approach is important to avoid a misdiagnosis of Li-Fraumeni syndrome.

## Case Report

A 66-year-old male former smoker with an alcohol abuse disorder presented to a Brazilian tertiary care center with a history of intermittent colicky abdominal pain and changes in bowel movements. He had nausea, vomiting, and severe constipation. The patient was submitted to an emergency surgery 1 month following the onset of symptoms due to a small bowel obstruction. The pathology report from the segmental small bowel enterectomy revealed a multicentric ­well-differentiated neuroendocrine tumor (NET) invading the sub serosal layer (19 lesions measuring up to 1.2 cm). Angiolymphatic invasion was also observed. The resection margins were disease free. Five of 7 mesenteric lymph nodes were compromised with metastatic NET but did not have extra-capsular dissemination. Immunohistochemistry was positive for synaptophysin (diffuse pattern), chromogranin A (diffuse pattern), serotonin, CK35BH11, and CDX2 (focal pattern). Tumor cell division was less than one mitosis for every 10 consecutive high-power fields (mit), and the proliferation index (Ki67) was less than 2% (grade 1). A computed tomography scan of the chest, abdomen, and pelvis revealed no evidence of metastatic disease.

The patient reported a personal history of prostate cancer diagnosed at the age of 61 years, treated with radical prostatectomy. He also reported a familial cancer history with 3 of 4 siblings affected by cancer at unknown ages, including breast, prostate, and head and neck cancers, one aunt (breast cancer) and her son with metastatic cancer (unknown primary site).

After 3 years of follow-up post NET diagnosis, the patient was enrolled in an investigational research protocol which started recruiting non-syndromic patients with gastroenteropancreatic NETs to be screened for germline mutations in genes associated with NET-predisposing syndromes.^1^ The patients provided written informed consent for participation in the trial, data collection, and publication of clinical and molecular data. The study protocol was approved by the Ethics Committee of Hospital Sírio-Libanês (HSL2015-15), and the Instituto do Câncer do Estado de São Paulo (NP762/15).

## Molecular Tumor Board Review

DNA was first obtained from peripheral blood leukocytes. The DNA libraries were generated using TruSeq Custom Amplicon (Illumina) and sequenced on MiSeq (Illumina), a Next Generation Sequencing (NGS) platform, to cover the coding regions of the *FLCN*, *MEN1*, *RET*, *NF1*, *TP53*, *TSC1*, *TSC2*, and *VHL* genes. The sequences were aligned to the human reference genome (GRCh37). Single-nucleotide variants and insertion-deletion mutations (indels) were identified using GATK.^2^ Only variants with a predicted damaging impact on protein function determined by the SIFT web server^3^ or by Polymorphism Phenotyping v2, PolyPhen-2,^4^ and a minor allele frequency lower than 0.1% according to the gnomAD dataset^5^ were used for further analysis.^1^

The patient harbored a missense variant in the *TP53* gene NM_000546.6 (TP53): c.413C>T (p.Ala138Val), rs750600586, with a variant allele frequency (VAF) of 22%. This variant is located in the DNA-binding domain and has not been previously reported in the gnomAD dataset.^5^ According to the Clinical Variant Database (ClinVar),^6^ this variant has been reported by a single submission, Ambry Genetics, as likely pathogenic. The amino acid alteration was predicted to be deleterious by In silico analyses (Revel score 0.87). Functional biological assays have described the *TP53* p.Ala138Val v ariant as partially functional, indicating reduced gene activity.^7^ This variant meets the ClinGen Resource.^8^ The *TP53* Expert Panel Specifications and ACMG/AMP Variant Interpretation Criteria of Pathogenicity PM2 support moderate/PP3/PS3 and were consistent with a variant of uncertain significance.^7^ A summary of variant characteristics is presented in Table 1.

**Table 1.** Variant information.

Genetic alteration	Chr17: 7675199 G> A (GRCh38) Chr17: 7578517 G> A (GRCh37)
Preferred name	NM_000546.6(TP53):c.413C>T (p.Ala138Val)
Exon	55 (NM_000546.6)
Protein domain	p53 DNA-binding domain
dbSNP reference	rs750600586
Revel	0.87
Allele frequency (gnomAD v2.1.1)	Absent
ClinVar	Likely pathogenic (1)
ACMG TP53 specifications	Uncertain significance (PM2supporting/PP3/PS3moderate)
Cases reported in literature	Two sisters confirmed to carry the variant: (1) breast (33 years-old); (2) breast (33 years-old) + uterine leyomiosarcoma (43 years-old)+ stomach. Other siblings were diagnosed with (3) breast (34 years-old); (4) gluteal tumor; (5) colorectal; (6) his daughter had “brain tumor” Breast tumor FFPE confirmed LOH of the wild type allele^9^

Numbers in brackets refers to the cases of sisters and siblings reported in literature.

**Figure 1.**
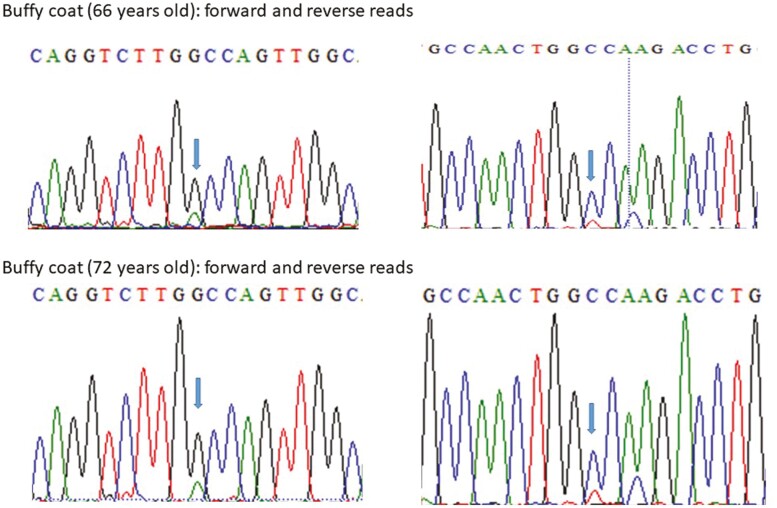
Sanger sequencing with forward and reverse primers indicating the *TP53* p.Ala138Val variant with low allele fraction in blood drawn at the ages of (**A**) 66 years and (**B**) 72 years.

**Figure 2.**
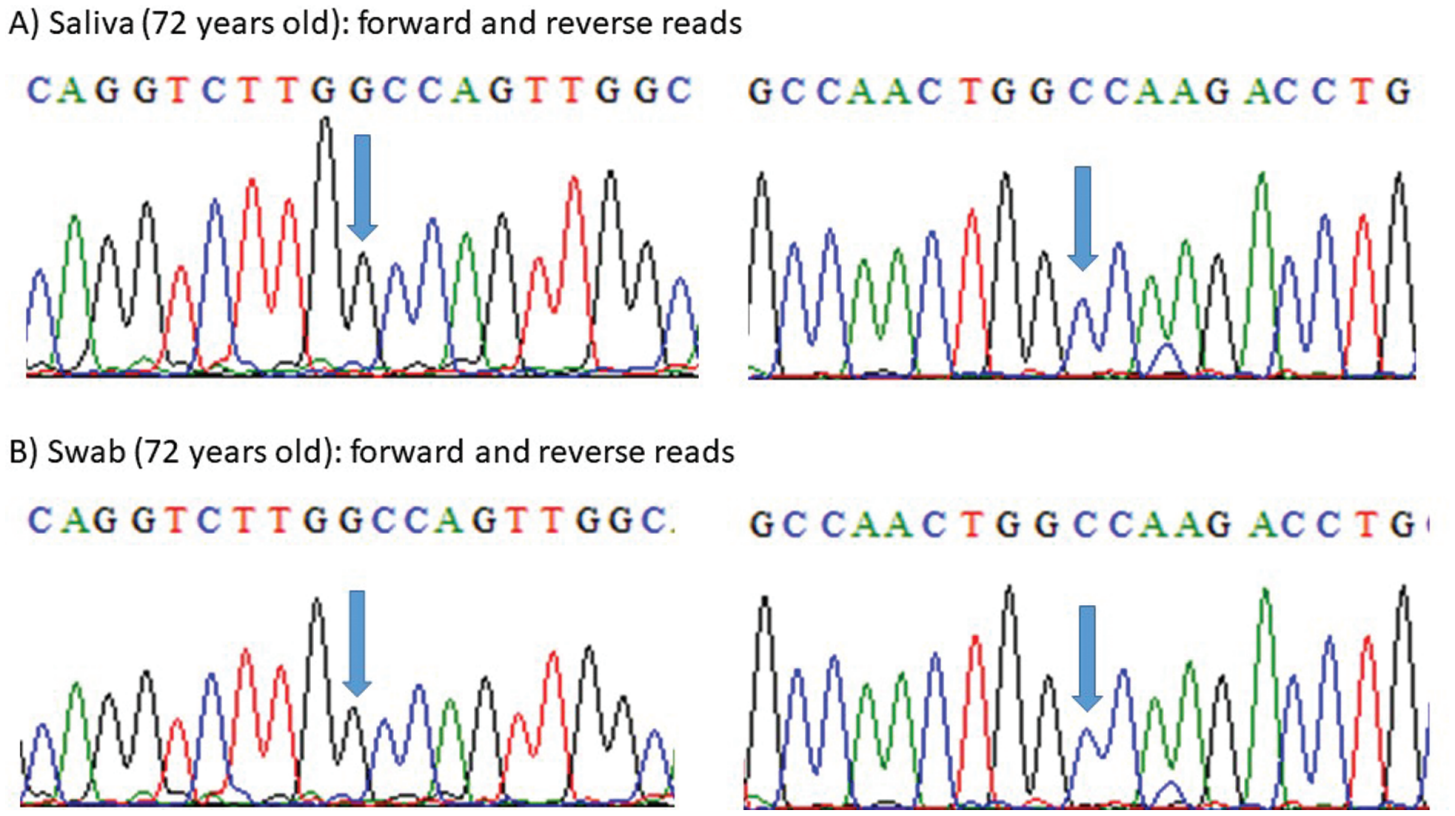
Sanger sequencing with forward and reverse primers indicating the absence of the *TP53* p.Ala138Val variant in samples collected at the age of 72 years in (**A**) saliva and (**B**) swab.

Additional tests were performed to investigate the origin of the variant. To validate the presence of the variant, an orthogonal method (Sanger sequencing) was performed using the same sample. Manual analysis of the chromatogram showed a significant difference in the peak height corresponding to the C and T alleles, corroborating the previous finding of a low VAF variant (Fig. 1A). We recontacted the patient to collect a new blood sample, oral swab, and saliva samples for DNA extraction and *TP53* variant testing. We confirmed the presence of the *TP53* p.Ala138Val variant in the DNA extracted from the blood, as well as differences in the peak height corresponding to the C and T alleles (Fig. 1A and 1B). However, this variant was not detected in the oral swab and saliva samples (Fig. 2A and 2B).

## Discussion

Germline pathogenic variants in *TP53* are associated with Li-Fraumeni syndrome (LFS), an autosomal dominant cancer predisposition syndrome associated with early onset cancers, including adrenocortical cancer, brain cancer, breast cancer, sarcomas, and other cancers.^10^ While some investigations reported a lifetime risk of malignancy of nearly 100% by age 70, these studies were based on individuals with a personal or familial history of cancer.^11^ Recent reports have demonstrated that the prevalence of potentially pathogenic germline variants of TP53 may be as high as 0.2% in the general population, suggesting lower penetrance of the syndrome and a wider phenotypic variability.^12^

Here, we report a case of a patient with a personal history of 2 primary cancers and a positive family history of cancer, who harbored a likely pathogenic variant *TP53* variant with low VAF in a blood sample. Initially, this result could be misinterpreted as a Li-Fraumeni case. Nevertheless, considering the low VAF of the variant and a cancer history that did not meet Li-Fraumeni criteria, other tests were conducted to elucidate the case.

In fact, *TP53* is among the 5 most frequent genes found to be associated with clonal hematopoiesis (CH).^13,14^ Approximately 20% of commercial laboratory multigene panel tests reports of pathogenic/likely pathogenic variants in *TP53* represent aberrant clonal expansion rather than a true germline finding.^15,16^ Acquired mutation patterns in hematopoiesis can result in dominance of a subset of hematopoietic stem and progenitor cells. This phenomenon is called CH.^17^ CH is commonly related to aging chronic inflammation, smoking, and other chemical stressors and may confer a higher risk of hematological malignancy and death owing to cardiovascular disease.^17^ CH can also be observed in healthy patients.^18^ In addition, constitutional somatic mosaicism, a post-zygotic event during embryogenesis, could be another hypothesis to explain the present case. Mosaicism may occur in early embryonic development, so that tissues and organs derived from the mutated cell, from the same embryonic layer, share that specific mutation. This process can also occur in later developmental stages, in a more differentiated cell, so that similar tissues, such skin, may present with only a patch carrying the mutation.^19^ Here, we collected a second blood sample 6 years after the first DNA analysis, as well as buccal swab and saliva, 2 other common sampling methods. These noninvasive biological materials contain squamous epithelial cells (of ectodermal origin) and blood leukocytes (of mesodermal origin), but in different proportions.^20^

Considering the patient’s age, lifestyle, personal and family history of cancer, and the fact that the *TP53* variant was not detected in non-blood samples, the most compelling evidence leads to the indication of CH. However, testing other tissues (eg, fibroblasts, hair follicles, tumor) using NGS which is a technique that has a higher sensitivity than Sanger sequencing to detect variants with VAFs <10% could help to rule out in absolute the possibility of mosaicism. The possibility of hematological malignancy could also be raised, but the patient had no abnormalities in blood counts within 6 years of follow-up.

## Conclusion

This report intends to use a real life case to illustrate a potential pitfall from multigene panel tests reporting a likely pathogenic variant in *TP53*. If a pathogenic *TP53* variant with low VAF is detected by multigene panel during germline testing, an investigation should be performed to assess the possibility of CH or constitutional mosaicism. One interesting finding was that the cell population harboring the variant remained stable even after 6 years, suggesting the prospect that sequential measurements could be potentially useful for surveillance of leukemia development. In terms of clinical guidance, this multidisciplinary molecular tumor board evaluation spared the patient from unwarranted screening procedures.

## Data Availability

The data underlying this article cannot be shared publicly for the privacy of individuals that participated in the study.
